# Considerations for implementing regulation of decapods in science

**DOI:** 10.1017/awf.2024.50

**Published:** 2024-11-27

**Authors:** Adam Powell, Ann-Lisbeth Agnalt, Kevin Heasman, Amaya Albalat

**Affiliations:** 1 Department of Life Sciences, Aberystwyth University, Ceredigion SY23 3DA, UK; 2 Institute of Marine Research, Nordnesgaten 50, Bergen 5005, Norway; 3 Cawthron Institute, Nelson, New Zealand; 4 Institute of Aquaculture, University of Stirling, Stirling FK9 4LA, UK

**Keywords:** animal welfare, crustacean, legislation, research, sentience, 3Rs

## Abstract

Decapod crustaceans, commonly utilised for pure or applied scientific research and commercial food production, have generally remained outside ethical debate. However, in the last decade many parts of the world have seen an increase in public interest in the welfare of decapod crustaceans and statutory legal protection has been introduced in several countries. Although still limited to a small number of countries and remaining relatively unharmonised, relevant legislation could be increasingly broadened to include decapods in further jurisdictions. Much existing legislation, originally intended for protecting terrestrial vertebrates during scientific study, might be unsuitable for aquatic invertebrates such as decapods. Indeed, precedence with many fish species and cephalopods suggests detail is lacking with respect to fundamental guidance. Therefore, similar inclusion of decapods into such legislation could make welfare or scientific goals more challenging to achieve unless relevant guidance is available, particularly to animal care practitioners. This horizon paper aims to summarise existing decapod legislation, and the considerations required should decapods be included in current conceptual frameworks and scientific legislation.

## Introduction

The order Decapoda includes commercially fished and farmed crustaceans, consisting mainly of aquatic species such as crabs, lobsters, crayfish, prawns and shrimp. The increase in human population, living standards and associated longevity have accelerated the demand and commercial production of decapods (Stentiford *et al.* 2012; Jennings *et al.* 2016). More specifically in scientific research, decapods are widely studied in many fields encompassing biotechnological, medical and ecotoxicological research and development (Hamed *et al.* 2016; Vogt 2018; Passantino *et al.* 2021), ecological studies including contemporary issues such as climate change and microplastic pollution (Toh et al. 2022; Yin *et al.* 2022) and teaching (Cooper *et al.* 2022; Wallis 2023).

Globally, human use of vertebrate animals is regulated according to standard veterinary, agricultural and husbandry practices, which as a minimum require basic husbandry and maintenance of animals during commercial operations. Many countries further regulate the use of non-human vertebrates during scientific research procedures (Codecasa *et al.* 2021). In the UK, for example, the Animal Scientific Procedures Act 1986 (ASPA) regulates the use of protected animals (any living vertebrate other than humans, and any living cephalopod) during experimental procedures that cause pain, suffering, distress or lasting harm (PSDLH), thus impacting physical, mental and social well-being, including disease, injury and physiological or psychological discomfort (UK Government 1986). Decapods have generally been excluded from ethical debate and relevant welfare regulations (Passantino *et al.* 2021; Rowan *et al.* 2021).

A landmark review by Birch *et al.* (2021) analysed several criteria which cumulatively attributed evidence of decapod sentience (the capacity to have feelings). Whilst not individually conclusive, criteria included brain morphology, nociception (rapid detection and response to noxious stimuli) and more complex phenomena such as behaviour and learning. In relation to ASPA, frameworks need to clarify whether decapods can perceive PSDLH to help inform relevant legislation, perhaps via studies that investigate longer-term biological phenomena and wider criteria (Passantino *et al.* 2021). Observational studies on behaviour, learning and strategy (e.g. injury-directed activities, motivational changes) are likely to increasingly suggest the ability of decapods to perceive pain (Elwood 2022; Barr & Elwood 2024). Whilst from a scientific standpoint the debate on sentience continues (Briffa 2022a; Diggles *et al.* 2023), a working precautionary approach, alongside changing governmental policy, has meant that several countries (including the UK, under The Animal Welfare [Sentience] Act 2022 [AWSA]), have now recognised decapods as sentient beings (Birch 2017; UK Government 2022a; Wickens 2022).

The European Union (EU) also recognised all animals as sentient beings within founding agreements such as the Lisbon Treaty (EU 2007; Rowan *et al.* 2021). EU agencies additionally recommended extending scientific legislation to include decapods (European Food Safety Authority [EFSA] 2005). Whilst this remains to be implemented, regulatory precedence has been set outside the EU, with several countries incorporating invertebrates within relevant national legal frameworks. Specifically for decapods, this includes Norway, Switzerland and New Zealand (Smith *et al.* 2013; Passantino *et al.* 2021). Whilst AWSA does not cascade to specific legislation such as ASPA, it mandated the formation of an Animal Sentience Committee, responsible for analysing potential negative welfare impacts on sentient animals that may arise from government policy (UK Government 2022a). Consequently, a consultation process was initiated in 2023 to detail decapod use in science, potentially altering the future scope of UK legislation (UK Government 2022b).

Recent efforts to improve welfare in commercial sectors, for instance optimising husbandry and euthanasia (Albalat *et al.* 2022; Neil *et al.* 2024) are pertinent to decapod use in the research sector. However, consideration of how decapod welfare could be introduced within existing scientific governance frameworks remains lacking. It therefore seems timely to expand the discussion further, firstly by reviewing extant invertebrate legislation and precedence, and secondly, by considering decapod-specific aspects that would be relevant within current vertebrate-centric legislation.

## Ethical considerations

No animals were required for this desk study.

## Precedence for invertebrates within scientific legislation

Animals are referred to as ‘protected’ under scientific regulation, although this varies between countries or jurisdictions, taxon and life stage (Codecasa *et al.* 2021). EU Directive 2010/63 (on the protection of animals used for scientific purposes) is arguably the most inclusive and extensive legislation, harmonising scientific use of animals across all EU member states (EU 2010). As with many other legal frameworks, relevant EU and UK law also protects non-human vertebrates and cephalopods from early developmental stages (Codecasa *et al.* 2021).

Additional, specific inclusion of decapods within welfare legislation exists in several countries (Passantino *et al.* 2021). However, the spectrum of protected taxa varies between neighbouring countries, for example in Asia, with decapod protection during science and teaching mandatory in Thailand and Indonesia (Law of the Republic of Indonesia 2009; Animals for Scientific Purposes Act BE 2558 2015 [Retnam *et al.* 2016]; Wallis 2023). Even regionally, protected species may vary, for example, across Australian territories (Victorian Government 1986; Wallis & Katayama 2022). Delineation between general commercial and scientific use also varies in legislation. For example, both purposes are contained within the same law in Norway, which protects decapods from the time they start feeding as larvae (Norwegian Government 2009; National Research Ethics Committee 2015). On the other hand, legislation is split across two Regulations and Acts in New Zealand, which protects decapods after larval developmental stages have been completed (NZ Government 1999, 2018). The most detailed legislation, covering both general and scientific governance, is contained within Swiss legislation (Swiss Federal Council 2005, 2008; Eggel & Camendzind 2020; Swiss Federation 2020), although neighbouring Austria stipulates standards for general husbandry only (Austrian Federal Chancellery 2004). In summary, this simple retrospective suggests that decapod protection has proceeded in a relatively piecemeal fashion and is not harmonised according to purpose, practical detail, and protection status.

Furthermore, previous regulatory change has not always been logical. As an example, ASPA amendments in 1993 extended protection (from exclusively vertebrates) to one invertebrate species in the UK, the common octopus (*Octopus vulgaris*) (UK Government 1993). However, other cephalopod species, including the more prevalent curled octopus (*Eledone cirrhosa*) (Barrett *et al.* 2023) were not protected under ASPA and researchers were not obliged to record and submit numbers enrolled in scientific research. In 2013, the UK transposed EU Directive 2010/63/ into ASPA, which broadened protection to all cephalopod species (EU 2010; Dunn 2021). This demanded swift dissemination of available technical knowledge regarding cephalopod husbandry and associated legal obligations (e.g. Andrews *et al.* 2013; Smith *et al.* 2013). Nevertheless, specific, yet fundamental recommendations for cephalopods (for example, husbandry and euthanasia) are not specified in EU Directive 2010/63 (EU 2010).

## The 3Rs and decapods

The use of animals in research is driven by internationally established principles of utilitarianism and the 3Rs (Replacement, Reduction and Refinement; Russell & Burch 1959; Anon 2012). The concept is enshrined within ASPA, EU Directive 2010/63 and other national laws which specify animals as non-human vertebrates and cephalopods (UK Government 1986; EU 2010; Codecasa *et al.* 2021). Prior to discussing specifics of legislation in detail, a more fundamental question is whether the 3Rs approach would remain a suitable platform to promote decapod welfare and improve data integrity.

Replacement promotes alternatives to using protected animals, either partially or fully. The use of *in vitro* systems and *in silico* modelling may be useful alternatives to assist Replacement (Liu *et al.* 2019; Passantino *et al.* 2021). Whilst some *in vitro* research has been developed for decapod research, there are no available invertebrate cell lines, due to high taxonomic diversity, fragmented research effort and additional knowledge gaps in cell metabolic requirements (Domart-Coulon & Blanchoud 2022). Therefore, research is needed in this area to support Replacement within the decapod research field.

Reduction advocates using minimal numbers of animals whilst maintaining worthwhile and robust scientific data. Reduction encompasses universally applicable goals of optimal experimental design, associated robust statistics and avoidance of study duplication (as exemplified by the ARRIVE and PREPARE guidelines; Smith *et al.* 2018; Percie du Cert *et al.* 2020). In terms of minimising animal numbers at the planning stage, decapods may be well suited. Many species are aggressive, cannibalistic and naturally solitary (Romano & Zheng 2017). In captivity, the welfare obligation to maintain decapods separately, rather than communally, also creates a powerful (*n*) number of individual experimental units, promoting robust statistical study design. Although not a widely cultured taxa, modular rearing systems are commercially available for certain decapod groups such as clawed lobsters (*Nephropidae*) (Hinchcliffe *et al.* 2022.

The impact of genetic variation and disease in the study population can also result in weak experimental data and inflate animal numbers to an unnecessary level. Ensuring a high health status and proven genetic lines (within designated breeding establishments) also supports Reduction. This is best achieved by establishing domesticated model species cultured with a closed lifecycle (i.e. full control over successive generations, breeding and health status, with straightforward husbandry requirements in captivity). For aquatic taxa in extant legislation, this only encompasses specifically zebrafish (*Danio rerio*) (EU 2010). In the case of decapods, whilst there is emerging interest in decapod veterinary care (e.g. Wahltinez *et al.* 2022), the lack of conventional immune memory in invertebrates (Rowley & Powell 2007) suggests that preventative measures supporting Reduction, such as vaccination, would confer limited benefit or proven pathogen resistance. Nevertheless, a general biomedical model may lie with crayfish species (Mykles & Hui 2015; Vogt 2018), whilst hermit crabs have been suggested for behavioural, and indeed sentience research (Briffa 2022b; Elwood 2022).

Refinement demands general and specific technical knowledge to optimise the lived experience of research animals and working to ensure that they have a good life. Additionally, this supports satisfactory data quality during scientific procedures. Refinement includes exemplary husbandry, positive welfare, seeking minimally invasive techniques, humane endpoints, and pain control (Anon 2012). However, our understanding of wild decapods is limited, in terms of maintaining them in their preferred environment in captivity, potentially over long periods of time. For instance, maintaining a robust and commonly studied European decapod (shore crabs [*Carcinus maenas*]) in captivity for a six-month period had a detrimental effect on their health, despite provision of husbandry and aquarium conditions that were hitherto deemed satisfactory (Wilson *et al.* 2022). Encouragingly, behavioural assessment techniques have been adapted to support positive welfare in captive decapods (Narshi *et al.* 2022).

Sharing expertise and developing best practice protocols are clearly needed, and transferable knowledge from decapod farming could support Refinement. Recent advances include species-specific operational welfare indicators, for example in abundantly farmed penaeid shrimp (e.g. the Pacific whiteleg shrimp, *Penaeus vannamei*) (Albalat *et al.* 2022; Pedrazzani *et al.* 2023) and continuous remote monitoring systems resulting from the emerging field of precision aquaculture (Browning 2023). Nevertheless, there is a lack of available veterinarian expertise, or consensus surrounding aquatic invertebrate health, disease diagnosis, treatment or euthanasia (Wahltinez *et al.* 2022). There remain several opportunities and needs to establish and improve anaesthesia, ethical killing and less-invasive sampling methods during research (Rottlant *et al.* 2023; Crump *et al.* 2024). Refinement will also require further research or ethical debate surrounding detrimental practices, for example claw banding and particularly ‘nicking’ (Johnson *et al.* 2016). While both practices compromise animals displaying normal behaviours, in some cases banding might be required to prevent physical trauma caused by intraspecific aggression.

Whilst there has been some call to revise or redefine the 3Rs for contemporary use (Tannenbaum & Bennett 2015; MacArthur-Clarke 2018), considered use of conceptual frameworks would likely improve the care of decapods when utilised in science and teaching, or at least provide more accountability regarding the use of these animals for research purposes.

## Scientific legislation

The following section will discuss the extensive, albeit vertebrate-centric legislation that currently does not protect decapods (EU Directive 2010/63 and ASPA). Cumulatively, regulation of protected animals during research and teaching generally falls within four practical sections (UK Government 1986; EU 2010; Codecasa *et al.* 2021): Breeding and supply; care and accommodation; procedures that cause pain, suffering, distress or lasting harm; and appropriate euthanasia. There is also increasing emphasis on improving the psychological well-being of research animals (Englund & Cronin 2023).

### Defining when a decapod becomes a protected animal

The age (or, more accurately, life stage) at which aquatic taxa become protected under law is variable. Cephalopods are protected upon hatching while, for fish, it is at the point of independent feeding (EU 2010). Research involving early life stages of fish demand specific knowledge, experience, and awareness that experimental populations may transition into a protected status during a scientific study. Precise timing varies not only between any poikilotherm species but also correlates with culture temperature (degree days), with decapod species being no exception. Decapods possess a range of reproductive strategies, encompassing widely variable fecundity, life stage developmental forms and stage durations. For example, redclaw crayfish (*Cherax quadricarinatus*) may brood a few hundred embryos which remain attached and develop on the female abdomen, emerging as precocious benthic juveniles (Haubrock *et al.* 2021). In contrast, Pacific whiteleg shrimp (*P. vannamei*) release several hundred thousand embryos shortly after fertilisation, which following hatching and initial reliance on yolk, develop through twelve larval stages in a pelagic environment, increasingly foraging larger and more active prey (Scott-Quackenbush 2001; Food and Agriculture Organisation [FAO] 2007).

Although detailed decapod larval ecology is known for a limited number of species, a precautionary and straightforward approach (cephalopod model – protect upon hatching) could be beneficial and is practiced in Norway (Norwegian Government 2009). Alternatively, the study of larval species that initially lack mouthparts and remain lecithotrophic (nutritionally sustained by yolk reserves) following hatching would more reasonably fall under the fish model (protect upon first feeding). Further targeted scientific review focusing on larval life stages, and subsequent ethical debate, would need to agree on the stage at which decapods are likely sentient, and logically become protected.

Relevant legislation demands accurate records of animal numbers enrolled within project licences to populate publicly available national welfare audits (UK Government 2022c). Should decapods become protected, the potentially immense number of decapod larvae and juveniles could contribute significantly to published values, both for individual establishments and nationally, running contrary to efforts to reduce the numbers of animals in research (UK Government 2014a; Marshall *et al.* 2022). Accurate quantification of mortality at these stages will be challenging given the high fecundity and larval mortality associated with aquatic invertebrate reproductive strategies. Nevertheless, this is a known issue within the aquaculture hatchery sector with accurate counting devices under development (e.g. Li *et al.* 2023).

### Decapod supply

Breeders, suppliers and users of animals are regulated within scientific research legislation and are preferentially sourced from licenced breeding facilities (EU 2010) to assist Refinement. Zebrafish have been utilised as a biomedical and genetic model since the early 1980s (Streisinger 1981) and are the only fish species stipulated within EU legislation which must be sourced from a licenced breeding facility (EU 2010). All other fish species and cephalopods can therefore be obtained via alternative means.

Commercial decapod aquaculture has reached a sophisticated technological level for some species, such as penaeid shrimp (Barki *et al.* 2010; Castillo-Juárez *et al.* 2015). Provided the species of interest is farmed, stock can be secured via commercial aquaculture facilities which may possess specific pathogen health status. Often, the quantity required for small-scale research purposes is negligible compared to commercial supplies, and for a minor customer such as a research project manager procurement can be challenging (A Powell, A-L Agnalt, K Heasman, A Albalat, personal observation 2023). However, for most species closure of the lifecycle and genetic manipulation is uncommon.

Although the number of farmed species is very limited in comparison to overall species diversity, it is likely much research will concentrate on fished or farmed decapod species due to their commercial importance. Nevertheless, a significant proportion of research and teaching is likely to focus on species that are not commercially produced. Should they become protected, decapods taken from the wild require an exemption prior to use in science, with an obligation to capture specimens humanely and competently, and stipulations on ‘Setting free’ after use (EU 2010). Additionally, wild caught animals also have an unknown health status and genetic provenance or variation. Although no decapod is currently under CITES protection (Convention on International Trade in Endangered Species of Wild Fauna and Flora [CITES] 2024), national or regional regulations may restrict the species, number, size, location or method of capture; or furthermore, keeping or release of non-native and likely imported species.

For decapods, procurement from the wild could encompass a range of habitats and capture methods, most simply via field collection in person. However, much procurement would need to rely on commercial, wild-capture fisheries, encompassing active or static nets and traps. Varying degrees of physical damage, physiological stress and morbidity can occur depending on capture method and the quality of subsequent husbandry (Fotedar & Evans 2011; Stoner 2012). Transportation conditions that are sub-optimal for a particular species can elicit stress and morbidity and associated ethical and welfare concerns (Powell *et al.* 2020). Therefore, general and scientific EU legislation would need to consider if and how to regulate decapod procurement from the wild in a manner that is achievable in practical terms, whilst securing high welfare status before, during and after individual studies. For instance, emersed or iced transport of decapods for any purpose is banned in Switzerland (Swiss Federal Council 2008).

### Decapod care and accommodation

For vertebrates and cephalopods, it is a fundamental obligation to ensure satisfactory care and accommodation for stock and experimental populations of animals used in scientific research. This currently includes taxonomic group and species-specific requirements, such as detail on housing dimensions and stocking density for discrete mammalian, avian, reptile and amphibian groups (EU 2010). The legislation additionally states that the care, accommodation needs and characteristics of each species should be addressed, and ideally harmonised and updated as knowledge is developed.

However, legal requirements for fish, combining all species and life stages, are somewhat limited to maintaining ‘adequate’ or ‘appropriate’ aquatic environmental parameters, whilst there is apparently no guidance for cephalopods (EU 2010). Further information may be available via national codes of practice (e.g. UK Government 2014b), however the limited and unharmonised detail on specific animal care within European legislation is a challenge (Marinou & Dontas 2023) and adaption of the Five Domains model to aquatic animals remains to be formally established (Perkins 2021). Although it would be unreasonable for such documents to provide detailed specific advice pertinent to every species and life stage and provenance, standardised fundamental requirements for invertebrate care are needed, should decapods be included in future legislation.

Whilst decapods share many similar biological characteristics, the diverse anatomy, physiology and life history inevitably influences husbandry requirements. There are over 17,000 recorded species, inhabiting a range of marine, freshwater and terrestrial habitats (De Grave *et al.* 2023). Conservative estimates suggest that about 50 species are farmed, and generally possess contrasting species- and life stage-specific husbandry requirements (FAO 2022). Therefore, only a very small fraction (*circa* 0.3%) of known species is understood at a level that would confer knowledge to support care and welfare of decapods in captivity. For cultured species, commercial sensitivities may preclude dissemination of production manuals, although material is available via the public sector, for commonly farmed (e.g. tropical marine and freshwater shrimp; FAO 2002, 2007) and emerging species (e.g. clawed lobsters; Burton 2003; Powell *et al.* 2015). To the authors’ knowledge, there remains only one specific decapod laboratory handbook available (Ingle 1995; updated, Elwood & Ingle 2024) and a recent guidance document for decapods in research (Crump *et al.* 2024).

In addition to care and accommodation requirements, legislation requires adequate staff education, training and competence, encompassing variable responsibilities during scientific management. These include general competencies (designing and carrying out procedures, animal care, culling), and species-specific managerial responsibilities (overseeing procedures, providing species information and training; EU 2010). Furthermore, the requirement for suitable veterinary and unbiased welfare support, alongside competent inspections, would likely demand development of novel training, potentially incorporating basic health checks, husbandry and commonly used procedures. Such knowledge would also support competency within related animal welfare bodies and, indeed, ethical review panels could change or expand markedly (Cooper *et al.* 2022), commensurate with increased quantity, novelty and animal numbers realised in project proposals. External and internal management and governance, which may include training, examination and licencing at many levels, will be challenging to achieve with limited species knowledge and before formal guidelines have been agreed and established. To the authors’ knowledge, Swiss law is unique in that it stipulates a statutory need for decapod-specific training of personnel in correct handling, biology, water quality monitoring and housing (Swiss Federal Council 2008).

### Regulated procedures that cause pain, suffering, distress or lasting harm

Whilst planning and performing a regulated procedure, researchers have several pertinent obligations. These include: avoiding death as an endpoint; to classify procedure severity levels using assignment criteria; to reach decisions on continued or re-use of animals; and to report actual rather than predicted severity (EU 2010). In UK legislation, a regulated procedure means any procedure which may have the effect of causing the animal a level of pain, suffering, distress or lasting harm (PSDLH) equivalent to, or higher than, that caused by the introduction of a needle in accordance with good veterinary practices. This definition would also be applicable to decapods, albeit perhaps refined somewhat to reflect the highly calcified exoskeleton of many species. Furthermore, procedures may be assigned across four severity categories, with specific (vertebrate-centric) examples provided in the legislation (EU 2010). For example, procedures defined by an upper limit on blood sample volume, or duration of food withdrawal, would be challenging to apply to decapods, which have an open circulatory system, and are poikilothermic with potentially low energy expenditure.

Nevertheless, general assignment of procedure severity category may occur on a case-by-case basis within specific studies, based on animal life history, the nature and cumulative PSDLH caused by procedures, preventing natural behaviour, and humane endpoints (EU 2010). Should decapods become protected, the assignment process will require good understanding of potential welfare indicators that can be used to evaluate likely severity of any proposed procedure. These may be radically different, or indeed rather subtle, compared to vertebrates (Coates & Söderhäll 2021. For instance, cortisol is often used as a stress biomarker in vertebrates, however decapods rely on an alternative hyperglycaemic response system (Lorenzon 2005; Sadoul & Geffroy 2019) with serum glucose and lactate also likely established indicators of physiological stress (Conneely & Coates 2024).

Operational welfare indicators are often relatively simple, visual observations that infer the welfare state of an animal or population. Decapods show primary responses to stressors such as behavioural defensive postures, increased locomotion or shelter-seeking (Stoner 2012). Should these fail, the animal can release at least one appendage at a predefined breakage plane in the carapace (autotomy) to promote escape whilst maintaining homeostasis (Fleming *et al.* 2007). Therefore, autotomy could be used as a welfare indicator during regulated procedures. Alternatively, some behavioural changes have been recorded during the onset of morbidity (e.g. Brown crab [*Cancer pagurus*]; Barrento *et al.* 2012). These have been used to define vitality indices and include environmental parameters, creating reflex action mortality predictors (RAMPs) to predict morbidity and mortality (e.g. for the Norway lobster [*Nephrops norvegicus*] and blue crab [*Calinectes sapidus*] (Albalat *et al.* 2017; Walters *et al.* 2022). Further, decapod integument can change according to infection status, albeit in a limited number of diseases, such as shell disease or *Sacculina* spp infection (Shields 2012). Therefore, efforts could be made to extend these approaches to assist assignment of procedure severity.

### Anaesthesia and toward ethical killing

Appropriate methods of anaesthesia and ethical killing are fundamental within animal scientific legislation, encompassing pain control during severe procedures, stock (non-experimental) management, and to euthanase experimental animals during or following experimentation (EU 2010). Satisfactory protection of decapods under scientific legislation would therefore require extension of similar protocols. Whilst historically it has been challenging to confirm efficacious and ethical anaesthesia of decapods, due to their differing neural system anatomy, neurotransmitter repertoire and hard exoskeleton (Belanger 2005; Walters 2018), recent reviews of compounds and techniques used for decapod anaesthesia have been published (de Souza Valente 2022; Wahltinez *et al.* 2022) and a decision support tool is now available (Rottlant *et al.* 2023).

Ethical killing of decapods may occur following a scientific procedure, or be termed euthanasia (to end suffering), or slaughter (for consumption) across commercial, domestic and scientific sectors. However, existing methods for stunning and slaughter of decapods are varied (Yue 2008; Conte *et al.* 2021) with many considered inhumane by the EU (EFSA 2005). Under scientific legislation, appropriate euthanasia methods are taxon-specific, require training and are stated plainly for some taxa, such as fish but not cephalopods (EU 2010). Norwegian and New Zealand legislations require decapods to be rendered insensible or stunned before imminent destruction (Norwegian Government 2009; New Zealand Government 2018). Decapod euthanasia under Swiss legislation follows a detailed precautionary approach, demanding training and specifying electrical stunning prior to additional boiling, splitting or spiking, and differentiating the optimal method according to the particular body plans of the decapod group (Swiss Federation 2020). Data so far indicate that electrical stunning might be effective for some species (Roth & Øines 2010; Roth & Grimsbø 2013; Neil *et al.* 2022, 2024). However, further work is needed in this area, particularly in terms of confirming the level of insensitivity achieved using decapod-equivalent electroencephalogram (EEG) data and defining animal-based indicators that can be used as proxies of insensitivity by operators.

### Animal welfare implications

Decapod crustaceans are increasingly becoming the subject of welfare and ethical considerations during scientific research. Recent reviews on this topic have suggested that decapods are sentient beings. Existing legislation, originally designed to protect vertebrates during scientific research, could soon be broadened to include decapods. However, precedence with other aquatic animals and invertebrates (such as cephalopods) shows that inclusion into legislation is poorly supported in terms of fundamental requirements such as general care and euthanasia. Additionally, much of the terminology used in such legislation is not compatible with the general biology of decapods or suffers from a lack of knowledge. This horizon paper considers the challenges of adding decapods into extant scientific legislation, and potential ways forward to practically deliver improved decapod welfare and scientific research.

## Conclusion and ways forward

Whilst the concept of the 3Rs is applicable to scientific endeavour and associated welfare of decapods, this horizon paper has highlighted practical issues that could arise should decapods be included within extant legislation regulating animals in science. Whilst there are encouraging practical developments in scientific and veterinary fields (e.g. Refinement; culling) and transferable knowledge from commercial sectors (e.g. operational welfare indicators; precision aquaculture), there are important knowledge gaps remaining and a lack of best code practice from an animal welfare perspective. Indeed, for those countries that do protect decapods, disparity remains (for example, the developmental stage that decapods are protected, husbandry training and requirements, and precise method of euthanasia).

While it is uncertain whether decapods will be incorporated into active legislation, precedence, best practice and experiences from other nations may be worth considering. For example, in one Australian territory, licencing of scientific activities involving animals includes discrete fieldwork activities, such as teaching, in addition to on-premises and breeding licences (Victorian Government 1986; Timoshanko *et al.* 2016; Wallis 2023). Further, a scientific code of conduct in Australia aims to harmonise standards, with varying degrees of joint self-regulation and enforced regulation between states. Defined as ‘co-regulation’, this governance approach could be a further method for scientists to reasonably ensure decapod care and welfare (Timoshanko *et al.* 2016). Research codes of conduct also aim to support ethical research using animals within and between Australia and New Zealand (Ministry for Primary Industries 2022; ANZCART 2024) and Malaysia (Wallis 2023). Decapod science may be considered niche in Norway, however care and welfare aims are supported and underpinned by collaboration between scientists, governing bodies, and the aquaculture sector (Norwegian Government 2009; A Powell, A-L Agnalt, K Heasman, A Albalat, personal observation 2023). Research organisations may also adhere to internal voluntary ethical standards surrounding decapod use which exceed national or territorial statutory laws, such as CSIRO in Australia (Rowe 2022).

From the *Discussion* in this horizon paper, inclusion of decapod taxa into scientific regulation needs careful thought if the aim is to significantly improve welfare. Collaboration between stakeholders, including scientists, governments and NGOs, will help ensure regulatory practicality and efficacy. This would preferably involve learning from the experience of other nations, and historical precedence, to harmonise any legislation. We hope that this overview underlines the points to consider should decapods be included in extant legislation and encourages government to consider research priorities to ensure maximum impact in any policy changes. This will foster better science whilst optimising animal care and welfare – the ultimate aims of the 3Rs and progressive scientific governance.
